# Structure of METTL3-METTL14 with an m6A nucleotide reveals insights into m6A conversion and sensing

**DOI:** 10.21203/rs.3.rs-3150186/v2

**Published:** 2024-10-23

**Authors:** Shan Qi, Abhay Kumar, Shuang Chen, Shuo Zhou, Manish Parihar, Carmen Villalobos, Navom Gupta, Siu-Hong Chan, Manjeet K. Rao, Stanton F McHardy, Shozeb Haider, Yogesh K. Gupta

**Affiliations:** 1 Greehey Children’s Cancer Research Institute, University of Texas Health Science Center at San Antonio, 8403 Floyd Curl Drive, San Antonio, TX 78229, USA; 2 Department of Biochemistry and Structural Biology, University of Texas Health Science Center at San Antonio, 7703 Floyd Curl Drive, San Antonio, TX 78229, USA; 3 Department of Pharmaceutical and Biological Chemistry, School of Pharmacy, University College London, London, United Kingdom; 4 New England Biolabs, 240 County Road, Ipswich, MA, 01938, USA; 5 Center for Innovative Drug Discovery, Department of Chemistry, University of Texas at San Antonio, San Antonio, TX, USA

**Keywords:** RNA modifications, m6A, Enzyme mechanisms, X-ray crystallography

## Abstract

The nuclear METTL3-METTL14 transfers a methyl group from SAM to convert the *N*^*6*^ of adenosine (A) in RNA to m^6^A and in ssDNA to 6mA. m^6^A marks are prevalent in eukaryotic mRNAs and lncRNAs and modulate their stability and fate in a context-dependent manner. The cytoplasmic METTL3 can act as a m^6^A reader. However, the precise mechanism during m6A writing, reading, or sensing is unclear. Here, we present a ~2.5 Å structure of the methyltransferase core of human METTL3-METTL14 in complex with the reaction product mimic, *N*^*6*^-methyladenosine monophosphate (m^6^A), representing a state post-catalysis but before the release of m^6^A. m^6^A occupies an evolutionarily conserved RNA-binding pocket ~16 Å away from the SAM pocket that also frequently mutates in cancer. We propose a two-step model of *swiveling* of target A upon conversion to m^6^A and *sensing* its methylation status by this pocket, enabling it to actuate enzymes’ switch from writer to an m^6^A-sensor. Cancer-associated mutations show impaired RNA binding dynamics, de-stacking, and defective m^6^A writing and sensing.

A heterodimer of Methyltransferase like-3 (METTL3) and its obligate partner METTL14 installs the majority of m^6^A (*N*^*6*^-methyladenosine) modification within the consensus DRACH (D=A/G/U, R=A/G, H=A/C/U) motif in eukaryotic mRNAs and lncRNAs^1–5^, chromosome-associated regulatory RNAs (carRNAs)^6,7^, and 7SK RNA^8^. METTL3 and METTL14 contain an MT-A70 family methyltransferase (MTase) core^9^ diverged from an ancestral β-class of bacterial MTases^10–13^. METTL3 hydrolyzes SAM to facilitate the transfer of its methyl group to the *N*^6^ amino group of the target adenine base in RNA *(in vivo* and *in vitro*)^1,4,9^ and ssDNA (*in vitro*)^11,12^. In contrast, catalytically deficient METTL14 stabilizes METTL3 and is thought to position RNA in METTL3’s active site^14–17^. m^6^A modifications modulate RNA stability and play essential roles in myriad biological processes, including but not limited to miRNA biogenesis, maintenance of neural stem cells, translation efficiency, transcription elongation, innate immune response, DNA break repair, circadian rhythm, and viral pathogenesis^18,19^. Consistently, severe growth defects observed in the cellular KO phenotype of METTL3 underscore METTL3’s essential role in maintaining cellular homeostasis during development^20–22^, cancer growth^23–26^, and viral infections, including SARS-CoV-2^27–29^.

While m^6^A deposition in mRNAs occurs in the nucleus and elevated METTL3 levels are associated with survival of acute myeloid leukemia^24,30^, non-catalytic functions of METTL3 outside the nucleus benefit lung cancer cells^23,25^. Cytoplasmic METTL3 can act as an m^6^A reader to promote the translation of mRNA of known oncogenes, thereby facilitating the crosstalk between m^6^A-bound METTL3 at 3’-end to the translation initiation machinery that has engaged the 5’- mRNA cap^23,25,31^.

m^6^A marks are also present in genomes of RNA viruses such as hepatitis C, Zika, dengue, West Nile, yellow fever, and SARS-CoV-2, and modulate viral replication and host immune response^27^. Thus, METTL3 has emerged as an attractive drug target for anti-cancer and anti-viral therapy development. Consistently, pharmacologic inhibition of METTL3 limits the growth of acute myeloid leukemia^26^ and SARS-CoV-2^28,29^. The first METTL3 inhibitor STC-15 that targets its SAM pocket has entered Phase I clinical trial (NCT05584111).

Despite significant advancement in the m^6^A field and interest in targeting it for therapy, the structural details of RNA recognition and catalysis by METTL3-METTL14 are lacking. Here we present a ~2.5 Å crystal structure of the methyltransferase core of METTL3-METTL14 bound to methyladenosine monophosphate (m^6^A), a product mimic of the methylation reaction (Fig. 1a). We show that m^6^A occupies a novel cryptic pocket constituted to a large extent by residues from METTL3 and an interface arginine (R298) of METTL14. This pocket is evolutionarily conserved in mammals, plants, and yeast (Fig. 1b). Importantly, residues that partake in interaction with m^6^A are mutated in gynecologic, stomach, kidney, and bladder cancers^32^ (Fig. 1b). When introduced into wild-type METTL3-METTL14, the mutant enzymes exhibit a significant loss in catalysis, perturbed RNA binding, and compromised ability of de-stacking of the target adenine for presentation to the active site. Our data suggest that the target base swivels ~120° after methylation for sensing by the cryptic pocket located ~16 Å away from the point of methyl transfer. METTL3-METTL14 uses this unique mechanism to sense the methylation status before releasing the substrate RNA. This arrangement will require de-stacking of the target base during catalysis and sensing. We also show that the wild-type METTL3-METTL14, but not the mutant, binds more tightly to an m^6^A-modified RNA to distinguish it from the unmethylated RNA. Moreover, the enzyme harboring R298P mutation, the most frequent mutation in endometrial cancer^32^, exhibits sub-optimal RNA binding, catalysis, and base de-stacking ability. Our results uncover entirely unexpected operating principles underlying methyl transfer and m^6^A-sensing by METTL3-METTL14.

## Results and Discussion

### Overall structure

**Figure 1:**
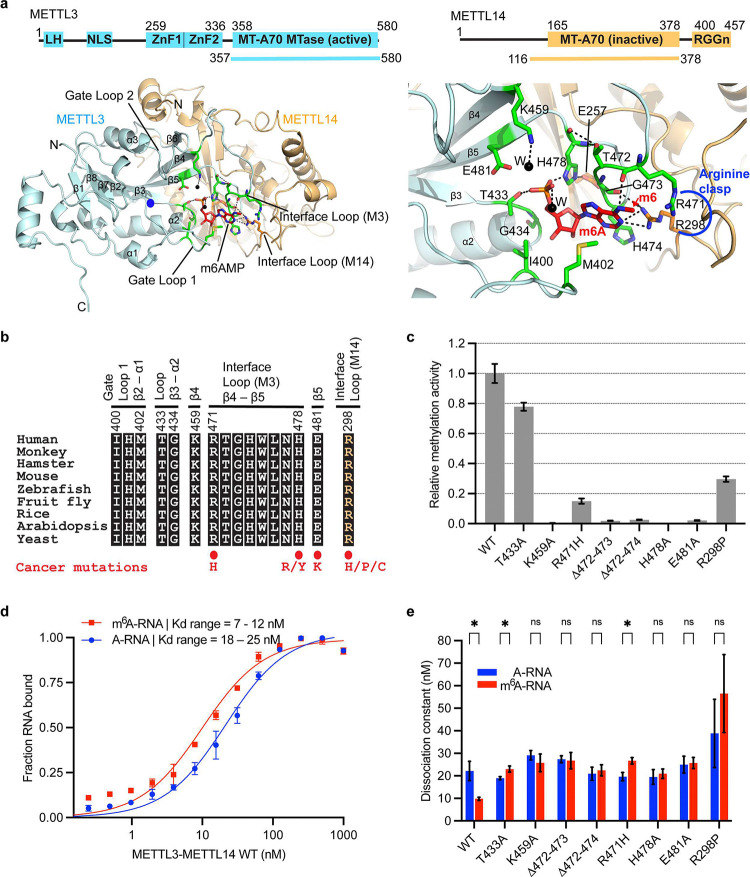
Structure of m6A bound human METTL3-METTL14 MTase core. **a,** METTL14; and boundaries of each used in crystallization are shown on top. Structure of the complex is shown in cartoon mode for METTL3 in cyan and METTL14 in orange; m6AMP (red) and interacting residues of METTL3 (green) and METTL14 (orange) are shown in stick mode. Blue dot, the position of *N*^*6*^ (in acceptor mode), i.e., ~3Å from the methyl group of the donor SAM. Methylated *N*^*6*^ of m^6^A is ~16Å away from its acceptor position in the catalytic pocket (blue dot). Black dots, water. Black dashes, h-bonds. The panel on right shows a close-up of the m^6^A interaction network, including the arginine clasp. **b,** An alignment of the regions participating in m^6^A con rms strict conservation of the interaction network throughout the evolution from yeast (Uniprot ID: P41833); arabidopsis (082486) and rice (Q6EU10); fruit y (Q9VCE6), zebrafish (F1R777), mouse (Q8C3P7), hamster (A0A1U7R3Z3), and monkey (A0A8J8YGJ7); to human (Q86U44). **c,** Methyltransferase activity results of full-length human METTL3-METTL14 (wild-type, WT) and eight mutant enzymes as derived from three independent experiments, with error bars indicating the range of data points from these experiments (n = 3). **d,** Quantitative measurement of RNA (red, m^6^A-RNA; blue, A-RNA) binding (n = 3) by the WT enzyme shown as binding isotherms fitted with a one-site specific binding model. The equilibrium dissociation constant or *K*_*d*_ derived for each mutant enzyme is plotted along with *K*_*d*_ of the WT enzyme (**e**). ns, not significant (*p* >0.05), * denotes *p* ≤ 0.05. Source data for panels **c-e** are provided.

The MTase cores of human METTL3 (aa 358 – 580) and METTL14 (aa 116 – 378) form an obligate heterodimer. METTL3 acts as an active SAM-dependent MTase, whereas METTL14, an inactive MTase, stabilizes RNA^14–17^. We co-purified the MTase core of METTL3-METTL14 from *E. coli.* We succeeded in resolving its structure in the presence of *N*^*6*^-methyladenosine 5’-monophosphate (m^6^AMP, referred to as m^6^A), a product of methylation reaction, by soaking m^6^A into apo crystals (Fig. 1 a, Extended data Fig. 1a-c). The difference omit map showed clear and unbiased electron density for m^6^A, which was refined well with no discrepancies for the ligand, surrounding regions, or the rest of the protein (Extended data Fig. 1d-g and table 1). METTL3-METTL14-m^6^A model was refined to ~2.5 Å resolution, with excellent stereochemistry and R_free_ and R_work_ of ~26.2 and 22.9%, respectively (Extended data Table 1). The final model contains one molecule each of METTL3 (aa 369 – 579), METTL14 (aa 116 – 402), one m^6^A, 90 water, and two ethylene glycol.

The overall fold of METTL3-METTL14 is similar to those reported previously^14–16^, except for notable changes in the region around the m^6^A binding pocket. MTases adopt a β-class of MTase fold with a central β-sheet of seven parallel and one antiparallel β-strands flanked by three helices on each side. Three major loops (gate loops 1 and 2 and an interface loop) emanating from the central β-sheet of METTL3 participate in SAM, RNA, and METTL14 binding. While the two gate loops exhibit high flexibility upon SAM or SAH binding and release, the interface loop remains rigid due to extensive protein-protein contacts from METTL14 MTase (Fig. 1a).

While the MTase core of METTL3-METTL14 is not catalytically active form, its structures derived by soaking SAM or SAH into these crystals provided crucial insights into the binding of methyl donor (SAM) and byproduct (SAH) and important conformational changes in the regions of METTL3 (gate loops 1, 2) surrounding these ligands^14–16^. The m6A-bound structure, which we report here, is the first nucleotide-bound form of the MTase core of METTL3–14 and provides crucial insights into the mechanism of this critical enzyme that is central to normal homeostasis and diseases. Since a small molecule targeting the catalytic pocket of METTL3 has entered human clinical trials for cancer therapy, our results showing the nucleotide binding in the regions outside of catalytic and SAM-binding pockets will pave the way for the rational design of more specific inhibitors.

### Evolutionarily conserved m^6^A pocket plays an essential role in m^6^A sensing

Strikingly, m^6^A occupies a cryptic pocket ~16 Å away from the methyl donor SAM pocket with its *N*^*6*^-methyl moiety in an energetically favored *syn* conformation, facing outward (Fig. 1a). Previously, this region was postulated to bind RNA due to its positive charge and polar nature^14–16^. m^6^A is stabilized by a vast network of specific interactions, mostly from METTL3 and R298 of METTL14. The purine ring of m^6^A is sandwiched between the side chain of M402 and the backbone atoms of the interface loop residues, R471, T472, G473, and H474. The two arginine residues (R471 of METTL3 and R298 of METTL14) act like a *clasp* to hold the *N*^*6*^-methyl moiety in place through a direct h-bond between R298 and *N*^*1*^, van der Waals and hydrophobic interactions between *N*^*6*^-methyl and its aliphatic portion, and the amino group of the R471 side chain, respectively. The carbonyl oxygen of G473 appears to neutralize the positive charge of the R298 residue. The carbonyl moiety of R471 embraces the *N*^*6*^ atom of m^6^A via a direct h-bond, while the opposite side is stabilized by the side chain of H474 via a π-π interaction. Altogether, the *arginine clasp*, interface loop residues R471-H474 and M402, forms a partial closure around the methylated purine ring of m^6^A. The ribose in the C3’-endo conformation is stacked between the backbone atoms of G473 and H478 and the side chains of I400 and H478. The phosphate group of m^6^A is locked in place by multiple direct h-bonds with its phosphoryl oxygens and side chains of H478, E481, T433, and K459 (water-mediated) – all from METTL3, and another water-mediated interaction with E257 of METTL14. The side chain of H478 holds the m^6^A phosphate on one side and E257 of METTL14 on the other, thus acting as a hinge (Fig 1a, Extended data Fig. 1g). Strict conservation of the extensive interaction network of m^6^A in human, animal, plant, and yeast suggests that m^6^A sensing by this cryptic pocket is an evolutionarily conserved mechanism (Fig. 1b). Several key residues that partake in m^6^A binding, such as R471 and R298 of the *arginine clasp*, E481, and H478 that stabilize the *N*^*6*^-methyl and phosphate groups are recurrently mutated in endometrioid and adenocarcinoma^32^ (Fig. 1b). We introduced the R298P mutation in METTL14, a recurrent mutation event in endometrioid carcinoma^32,33^, and the R471H, E481A, T433A, K459A, and H478A mutations in METTL3. In addition, we generated two deletion mutants (Δ472–473, Δ472–474) in which three residues of METTL3 (T472, G473, H474) that stack against the purine ring of m^6^A were deleted to shorten the interface loop.

We co-purified the full-length wild-type human METTL3-METTL14 and eight mutant enzymes from insect cells and probed their RNA methylation and binding activities. We used a 30-mer RNA oligo (NEAT2*) consisting of one canonical GGACU motif. Consistently, R298P and R471H mutants significantly reduced (up to 85%), whereas T433A resulted in ~20% loss in methyltransferase activity, agreeing with the reduced m^6^A levels observed in endometrial tumors harboring the R298P mutation^33^. The other five mutations in METTL3 (Δ472–473, Δ472–474, K459A, E481A, and H478A) completely abolished the RNA methyltransferase activity of METTL3-METTL14 (Fig. 1c). Thus, the evolutionarily conserved m^6^A binding pocket is essential for efficient conversion of A to m^6^A.

Next, we quantitatively determined the binding affinities of wild-type (WT) and mutant enzymes to the substrate and a product RNA, wherein the target A base within GGACU is replaced by m^6^A. We covalently attached a fluoresceine moiety to the 5’-end of both the substrate NEAT2* (A-RNA) and product (m^6^A-RNA) RNAs and performed fluorescence polarization-based assays. The WT enzyme can still bind the m^6^A-RNA and A-RNA with high affinity (*K*_*d*_ = 9–20 nM) (Fig. 1 d,e), corroborating previous studies attributing a sort of m^6^A-reader function to METTL3 *in vivo*^23,25,31,34^. In contrast, the mutants, including R298P and R471H (both mutated in cancers and belong to the *arginine clasp* motif that stabilizes the m^6^A) exhibited a loss in binding affinity with varying degrees, with R298P showing weakest binding (Fig. 1e, Extended data Fig. 1h). Thus, inability to sense and distinguish m^6^A properly by the R298P mutation could result in total m6A and promote tumorigenicity and growth of endometrial tumors as observed previously^33^. The nanomolar affinity observed in this fluorescence polarization (FP)-based assay for mutant enzymes suggests a significant contribution of accessory motifs such as zinc fingers (ZnFs) of METTL3 and RGG repeats of METTL14 to RNA binding, especially the predicted bulged stem-loop structure of NEAT2* RNA (A-RNA). These motifs are intact in both the wild-type and the mutant enzymes. This could be one of the reasons for not observing the radical change in overall RNA binding as measured by the equilibrium dissociation constant (Kd) for A vs. m6A RNA in an FP-based assay. Moreover, the residue aligning the catalytic and cryptic pockets would interact with the substrate (A) and the product (m6A) nucleotide of the ‘DR**A**CH’ sequence during the pivoting of the base upon conversion to m6A, respectively. Even if the mutations in the cryptic pocket retain overall RNA binding dominated by domains flanking the MTase core (ZnFs and RGG) and the secondary structure of RNA itself (GGACU containing stem-loop RNAs show higher affinity), they could still influence the pivoting of the m6A base and its release after conversion.

**Figure 2:**
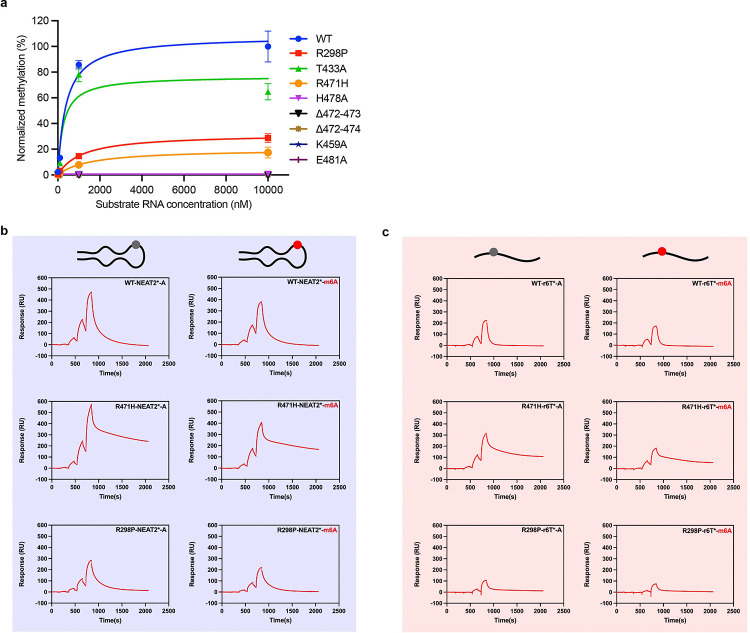
Enzyme and binding kinetics. **a,** Methylation of NEAT2* RNA by full-length METTL3-METTL14 (wild-type, WT and its mutants) at saturating concentrations of SAM and RNA. **b-c,** Kinetics of RNA binding to the WT and mutant METTL3-METTL14 as measured using surface plasmon resonance. Two RNA oligos (NEAT2* and a single-stranded RNA) comprising substrate A (grey circle) or product m6A (red circle) were probed.

We also performed a kinetic analysis by varying the concentration range of RNA substrate from 10 nM to 10 μM in the presence of a saturating concentration of SAM (10 μM) (Fig. 2a). Consistently, these results show that the wild-type enzyme yields the highest methylation activity, whereas the mutant enzymes show reduced methylation. Next, we studied the binding kinetics of the full-length METTL3-METTL14, wild-type (WT), and the two mutant enzymes harboring R298P and R471H mutations in METTL14 and METTL3, respectively. We employed the surface plasmon resonance (SPR) technique, a gold standard for studying binding kinetics that includes ON (K_on_) and OFF (K_off_) rates of RNA binding. We used our original RNA substrate, NEAT2 (30-mer bulged stem-loop) RNA, and its methylated version (NEAT2-m6A). We also examined a 14-mer linear RNA substrate (r6T) and its methylated form (r6T-m6A) to fully understand the kinetics of enzyme binding to GGACU-containing RNAs with different shapes and sequences. The SPR data of the WT enzyme fit well with a 1:1 binding model. As shown in Fig. 2b and Tables 1–3, the binding affinity and kinetics of METTL3-METTL14 enzymes differ significantly on the two RNA oligos tested. The structured NEAT2 RNA shows a 5-fold tighter affinity than a linear r6T substrate, while their methylated counterparts show reduced binding (1.3-fold for NEAT2-m6A and 2.3-fold for r6T-m6A), but the Kds (dissociation constants) are still in sub and low-micromolar range.

Interestingly, the ON (K_on_) and OFF (K_off_) rates for a linear substrate RNA differ 2–2.7-fold compared to NEAT2 RNA. A 2.7-fold faster OFF rate on a linear substrate would result in a rapid turnover and higher methylation of the GGACU motif residing in a linear RNA compared to the loop region of a stem-loop RNA. At the same time, the structured elements in RNA can help recruit METTL3-METTL14, as suggested by a 5-fold stronger affinity to NEAT2 RNA. These results suggest the RNA shape surrounding the core ‘GGACU or DRACH’ motif is a crucial determinant of methylation. This observation can explain, in part, why only a fraction of potential DRACH motifs (or perfect GGACU) are methylated *in vivo*.

The SPR data of the two mutant enzymes (R471H and R298P) revealed a change in Rmax and mode of binding. While the SPR data of the WT enzyme fit well with the 1:1 binding model, the mutant data could only fit well with a two-state reaction model (Table 3). The R298P mutation in METTL14 results in a moderate loss (1.4–1.6-fold) of binding to the stem-loop NEAT2* RNA (Kd=256 vs. 356 nM) and its methylated counterpart (Kd= 337 vs. 538 nM) or the linear r6T RNA (Kd=1360 vs. 2160 nM) but a significant loss (>7-fold) in RNA binding occurred for methylated linear r6T RNA (Kd=3120 vs 22900). On the other hand, the R471H mutant exhibits much slower dissociation after binding, thus hampering the enzyme’s turnover. These data suggest that the two arginines (R471H of METTL3 and R298P of METTL14) in the m6A pocket, which is cryptic in nature, are important for recognizing the methylation status, and the replacement of R298 to proline and R471 to histidine would negatively impact the methylation of the canonical GGACU motif. Of note, striking differences in the mode of binding – two-state binding for R471H/ R298P mutants vs. one-state binding of the WT enzyme – and their dynamics (ON and OFF rates) likely alter the retention time of the enzyme on RNA with varied shapes and sequences or residence time for m6A of these RNAs in the cryptic pocket. Consequently, multi-stage RNA binding and altered dynamics could alter specificity. An independent study by Zhang et al. reported that R298P mutation alters the enzyme’s preference from GGAC to GGAU^35^. In this context, our structural and binding data provides crucial and timely insights into the existence of a cryptic pocket, the importance of two arginines for methylation of the canonical GGACU motifs, and how mutations at these positions could alter the dynamics of RNA binding of METTL3-METTL14 and ultimately alter enzyme specificity.

To assess the dynamic impact of the mutations in METTL3-METTL14 on m^6^A binding, we conducted supervised molecular dynamics simulations (SuMD) and analyzed the distances between the center of mass (com) of the m^6^A and the product m^6^A binding pocket (defined by residues within 4 Å of m^6^A (see methods) over time for wild-type and each mutant, including T433A, K459A, R471H, Δ472–473, Δ472–474, H478A, E481A, and R298P. The wild-type METTL3-METTL14 complex displayed distances around 1.5 Å for most of the simulation time, highlighting the presence of m6A within the product m6A binding pocket (Extended data Fig. 3a and 4a). Furthermore, this also indicates that the product m^6^A binding pocket in the wild-type is well-structured to accommodate m^6^A securely and thereby maintain a stable binding environment (Extended data Fig. 3b). In the wild-type, interactions between H478, T472, and m^6^A are observed. Some additional interactions are also formed; for example, T433 and E481 form stable interactions with the phosphate group via a water molecule. A salt bridge between K459 and E481 helps to stabilize the water molecule (Extended data Fig. 4a). In H478A, which is no longer able to interact with m^6^A, the phosphate group becomes mobile and is unable to anchor to A478 (Extended data Fig. 4f) . In T433A and R298P mutations, the distance between the center of mass of m^6^A and the product m^6^A binding pocket mostly ranged from 2 to 4 Å, suggesting that these mutations, while impactful, do not entirely abolish m^6^A binding (Extended data Fig. 4b, c). Consequently, these mutants could retain m^6^A in the product m^6^A binding pocket for most of the simulation time. It is interesting to note that in the T433A mutation, in spite of the h-bond interaction between the phosphate group and the side chain of T433 is lost, the interaction between H478 and the phosphate group remains intact (Extended data Fig. 4c). Therefore, this T433A mutation does not significantly affect the binding of m^6^A. In the case of R298P (Extended data Fig. 4b), although the *arginine clasp* was broken, the phosphate group remains stabilized in the same position, making interactions with H478. Moreover, E481 in the R289P mutant can also contribute to stabilizing the phosphate group via a water molecule (Extended data Fig. 4b). However, in the E481A mutant, this water bridge is lost. Similarly, the mutation in K459A results in the loss of a salt bridge with E481, leading to localized destabilization (Extended data Fig. 4d, g). The resulting electrostatic repulsion between the negatively charged side chains of E481 and the phosphate group of m^6^A pushes the m^6^A out of the product m^6^A binding pocket (Extended data Fig. 4g). For the two deletion mutants (Δ472–473, Δ472–474), m^6^A immediately leaves the product m^6^A binding pocket after the removal of the constraint in the equilibration steps, highlighting the essential nature of these residues in maintaining the binding pocket’s structure (Extended data Fig. 4h, i).

### Base swiveling facilitates m^6^A sensing

**Figure 3:**
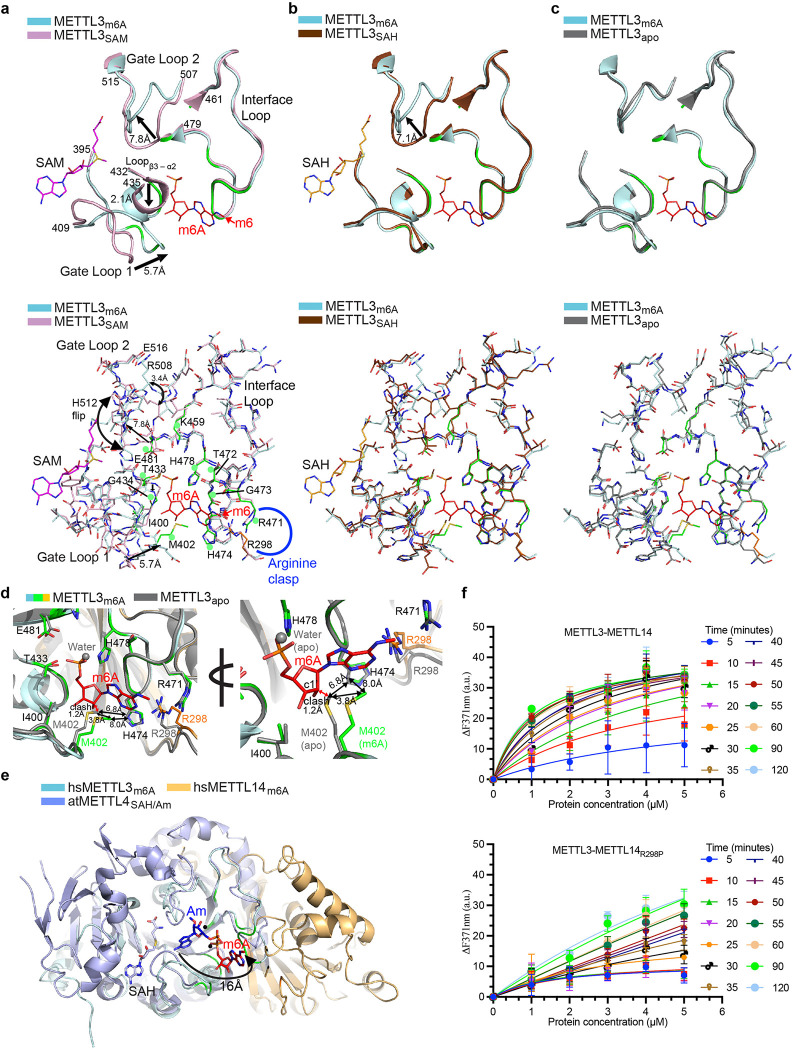
Base swiveling and loop orchestration. **a-c,** Upper panels show overlays of regions of METTL3 encompassing the catalytic motif, gate loops 1 and 2, and interface loop in METTL3 bound to m^6^A (red stick), SAM (pink stick), and SAH (orange stick). Arrows indicate the directional movement of loops. Lower panels: The entire region of each overlay is in stick mode. Green dots, the residues that form the m^6^A interaction network. **d,** Close-up of an overlay of m^6^A and apo MTase of METTL3-METTL14 shown in two orientations for clarity. The exit channel between M402 and H474 in the m^6^A bound conformation becomes wider (up to 8Å) to stabilize m^6^A and avoid steric clashes with its purine and ribose moieties. **e,** An overlay of MTase cores of arabidopsis METTL4 (light blue cartoons)/SAH (light blue stick)/Am (blue stick) and METTL3 (cyan)-METTL14 (orange)/m^6^A (red stick) clarifies the ~ 120° pivot of the base around phosphate. Black dots, water molecules in the m^6^A structure help stabilize the m^6^A and compensate for the loss in binding energy in the site emptied by base pivoting. **f,** Change in emission fluorescence intensity upon titration of increasing concentration of WT (upper panel) and R298P mutant enzymes (lower panel) with 2-aminopurine (2-Ap) containing RNA (n=3). See the methods section and source data for details.

The two loops in METTL3 (gate loops 1 and 2) surrounding the methyl donor SAM and acceptor base A pockets show varying degrees of flexibility upon SAM and SAH binding from their original positions in a ligand-free (apo) form^14–16^. Thus, we compared the m^6^A structure with three states (SAM, SAH, and apo). These loops also move in opposite directions upon m^6^A binding from their original positions in the SAM-bound METTL3 (Fig. 3a). Gate loop 1 (aa 398–409) moves ~ 5.7Å inward to the direction of m^6^A, whereas the gate loop 2 (aa 506–512) moves ~ 7.8Å outward, with several residues in this region, including H512, that flips ~180°. The invariant T433 and G434 from a small loop between β3 and α2 move ~ 2.1Å with the side chain of T433 rotating ~90° to stabilize the phosphate and ribose of m^6^A (Fig. 3a). This region remains unperturbed in SAH-bound METTL3, suggesting the m^6^A binding to this pocket occurs after hydrolysis of SAM (Fig. 3b). While the gate loop 2 in SAH remains in open confirmation, akin to SAM conformation, the position of gate loop 1 in m^6^A experiences significant repositioning of the M402 side chain (Fig 3c). Although m^6^A-bound METTL3 is most similar to the apo form with the smallest root mean square deviation for superposition of 1539 atoms of METTL3 achieved for apo (1.2), compared to SAH (1.5), and SAM (1.9), we observed notable changes in the m^6^A pocket (Fig. 3c).

The side chain of M402 from gate loop 1 in the m^6^A structure stacks over the purine ring of m^6^A. In the SAM-bound form, this region is moved >5Å away, but in the SAH and apo forms, the M402 side chain will sterically clash with m^6^A ribose (distance between C*ε* of M402 and C4’ of ribose ~1.2 Å). To avoid this clash, the side chain of M402 in m^6^A-METTL3 rotates > 45°, resulting in a ~3.8Å gain in the distance for the C*ε* atom compared to its position in the apo structure. As a result of this repositioning, the inter-gate area between interface loop (H474) and gate loop 1 (M402) becomes wider, from 6.8Å in apo to 8.0 Å in the m^6^A structure (Fig. 3d). Thus, gate loop 1 from one side senses the SAM and targets the RNA base at the point of catalysis (^395^DPPW^398^ motif). It then swivels after SAM hydrolysis to facilitate the sensing of m^6^A status of the target base at the opposite or *exit* site.

Another change occurs in how the side chain of invariant R298 (METTL14) orients within the *arginine clasp*. The R298 side chain rotates ~180° around its C_β_, although the guanidino group shifts slightly to form a direct h-bond with *N*^*1*^ of m^6^A (Fig. 3d, Extended data Fig. 1g). The orientation of the gate loops suggests that m^6^A-METTL3 represents a state of enzyme post-catalysis before release of any product or enzyme reset.

How does m^6^A swivel ~16Å from the point of catalysis to occupy this novel pocket in METTL3? To answer this question regarding the mechanism of base-swiveling, we once again employed supervised molecular dynamics (SuMD) simulations. The simulations successfully captured the transition of the m^6^A from the substrate A pocket to the product m^6^A binding pocket. The phosphate group of m^6^A makes h-bond interactions with H478 (Extended data Fig. 4a). This interaction acts like a hinge and anchors m^6^A, which then allows the nucleotide base in m^6^A to swivel and eventually occupy the product m^6^A binding pocket. Once in the product m^6^A binding pocket, the nucleotide base of m^6^A can form h-bond with R298 (Extended data Fig. 4a). The most stable structure of the m^6^A in the product m^6^A binding pocket displays a root-mean-square deviation (RMSD) of ~ 2 Å with the resolved crystal structure (Extended data Fig. 5). This close structural alignment with the crystallographic data highlights the significance of the observed transition mechanism and corroborates with the H478A mutation where the RNA methyltransferase activity is completely abolished in the METTL3-METTL14 complex. To further validate these interactions, we calculated the interaction energy between m^6^A and the METTL3-METTL14 complex (Extended data Fig. 5–7). The results showed significant binding affinity and stability, supporting the observed h-bond interactions and the overall structural integrity of m^6^A within the m^6^A binding pocket (Extended data Fig. 7).

To verify our findings, we superposed m^6^A-METTL3 over the structure of Arabidopsis METTL4, a member of the subclade of the MTA-70 family that possesses the substrate 2’-*O* methyladenosine (A_m_)^36^. The central β-sheet and the catalytic motif of the two enzymes (DPPW) overlay very closely. In this model, the acceptor *N*^*6*^ atom of Am resides at ~3Å or less from the methylsulfonium group of SAM for S_N_2 mechanism of direct methyl transfer. The phosphates of A_m_ and m^6^A lie in close proximity (~1.2Å). However, their purine and ribose rings are rotated ~120° in opposite directions, suggesting the base (A) pivots after conversion into m^6^A (Fig. 3e). Such a rotation may necessitate the de-stacking of the target base for its presentation to catalytic pocket and or base swiveling.

A water molecule at the putative site of the substrate A base is present in the m^6^A structure to compensate for the loss in binding energy in the emptied site by rotation of m^6^A from this site post-catalysis. This water coordinates with K459, and its mutation to alanine abolishes the methylation activity (Fig. 1c). SAM-dependent DNA methyltransferases, including the ancestral members of MTA-70 family MTases such as EcoP15I, efficiently flip the target adenine base out of the DNA helix into the catalytic pocket^37^. Although METTL3-METTL14 does not methylate dsDNA and dsRNA^11,12^, it can still de-stack the target base from a single-stranded RNA into the catalytic pocket, similar to the m^6^A/m^6^A_m_ eraser enzyme, FTO^38,39^, and the m^6^A reader, YTHDC1^40^. To test this activity, we replaced the target A (6-aminopurine) in a GGACU in a 14-mer ssRNA with 2-aminopurine (2Ap), a fluorescent nucleobase used as a conformational probe due to its high sensitivity to changes in the local environment induced by DNA^41^ and RNA MTases^42^. As shown in Fig. 3f, the change in fluorescence intensity (at 371nm) with increasing enzyme concentrations was rapid for WT but not the R298P mutant enzyme, confirming the diminished RNA binding and base de-stacking ability of the mutant enzyme. While the cellular impact of R298P mutation has been studied in the context of cancer^33^, our data now uncover a precise mechanistic role of R298 in m^6^A sensing. Thus, an elegant orchestration of loops surrounding the SAM/SAH, substrate A, and product m^6^A binding pockets enables m^6^A base swiveling and sensing (see Movie 1).

To capture an AMP-bound METTL3–14, which could serve as a control structure, we extensively attempted co-crystallization and soaking of AMP into apo crystals but could not observe AMP around the catalytic pocket (DPPW or motif IV) or the m6A binding pocket. We reason the highly mobile nature of the substrate adenine base in the catalytic pocket for this. To gain insights into this phenomenon, we examined the Am binding of the published structure of Arabidopsis METTL4 (PDB: 7CV6)^36^. The slightly low occupancy coupled with high average B-factors (B = 177 Å^2^) of Am base in Arabidopsis METTL4 suggest a highly mobile nature of the Am base in the catalytic pocket (Extended data Fig. 2a). Furthermore, Am’s RSCC (real space correlation coefficient) score in Arabidopsis METTL4 is 0.66. A value of RSCC score below 0.8 indicates a modest fit^43^. In contrast, the m6AMP in our hMETTL3-METTL14 methyltransferase core structure fits nicely into the electron density at full occupancy (1.0) (Extended data Fig. 1 d-f). Consistently, an RSCC score of 0.85 with low average B-factors (B = 70 Å^2^) confirms a relatively stable mode of m6A binding to hMETTL3-METTL14 (Extended data Table 1 and PDB validation report).

While our work was in preparation, Corbeski et al. reported crystal structures of METTL3-METTL14 MTase core in complex with synthetic bisubstrate analogs (BAs), wherein methyl donor SAM was covalently linked to the substrate adenosine. These analogs may represent a transition state during methyl transfer^44^. This study suggests that the substrate nucleotide-bound structures of METTL3-METTL14 could only be resolved when the *N*^*6*^ of adenosine was covalently attached to SAM via a 2–3 carbon linker. Interestingly, despite spatial restriction imposed by covalent linkage, the substrate adenosine moiety still exhibits significant flexibility within and across crystals obtained from soaking two different analogs, e.g., BA2 and BA4 (Extended data Fig. 2b, c). For example, the adenosine moiety of the covalent analog BA2 samples two different orientations; in a ‘buried’ conformation, it occupies the substrate pocket of METTL3 and interacts with E481 and K513, whereas in an ‘alternate conformation,’ it rotates ~90° and exposes to the solvent. In the BA4-METTL3–14 structure, the covalently attached substrate adenosine rotates into solvent-exposed orientation while the cosubstrate SAM remains rigid and occupies the SAM-binding pocket in both structures (Extended data Fig. 2b, c). Importantly, the movement of the adenosine in BA-analogs will be limited due to the covalent linkage to SAM, which naturally has a high affinity. However, the adenosine continuously tries to move in and out of the catalytic pocket. We believe these structures could serve as a control where adenosine is captured in the methyl acceptor state of the *N*^*6*^, precisely the way we modeled it by comparing the METTL4-Am structure. Moreover, the gate loop 1 and the interface loop are disordered in METTL3–14 bisubstrate analog structures, most likely due to the lack of stabilizing interactions. In contrast, these regions of METTL3–14 are well resolved in our m6AMP-bound structure due to their stabilizing interaction with m6AMP. Altogether, these observations suggest a highly mobile nature of the substrate adenine (A) base in the catalytic pocket.

The center of mass distance measurements in the simulations revealed that m^6^A consistently maintained the shortest average distance of ~1.5 Å with low variability, indicating a strong and persistent binding interaction. In contrast, other nucleotides exhibited greater and more variable distances, typically exceeding 2 Å and occasionally reaching up to 5 Å, especially AMP, CMP, and GMP, suggesting weaker and less stable interactions. AMP, CMP, and GMP showed the largest RMSD values, > 4 Å, indicating the least stability in their binding positions (Extended data Fig. 3b). This instability can be attributed to the inability of M402 to form hydrophobic interactions with the methyl group of m6A. In AMP, the loss of the methyl group prevents M402 from stabilizing the nucleotide. Although AMP can still form strong interactions with the complex, the RMSD plot indicates significant flexibility within the binding pocket. The results from SuMD are in excellent agreement with our experimental findings, which highlight the specific binding preference of the METTL3-METTL14-core for m^6^A.

### METTL3-METTL14 acts as an atypical m^6^A sensor

**Figure 4:**
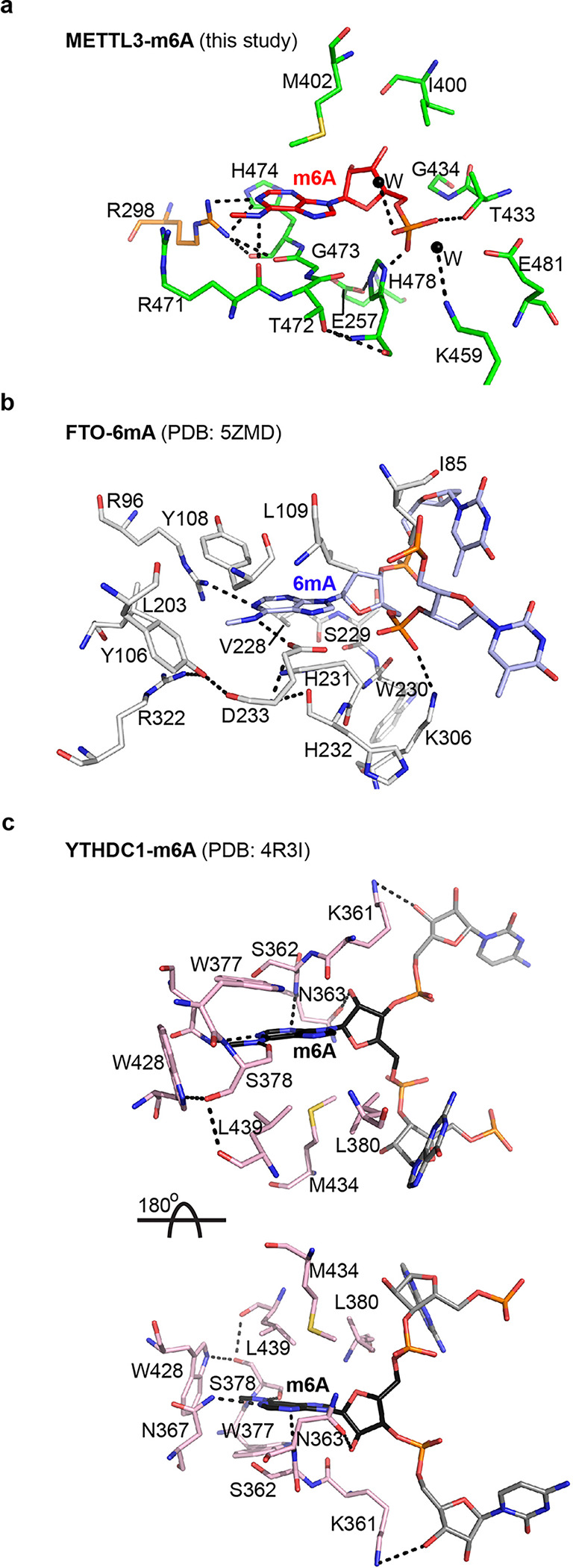
Mode of m A binding by writer/sensor, eraser, and reader. Interaction networks of m^6^A (red) binding to METTL3 (green), and METTL14 (**a**), 6mA (blue) binding to FTO (**b**), and m6A binding to YTH domain of YTHDC1 (**c**). The two nucleotides flanking the flipped methylated base in FTO and YTHDC1 are shown in light blue and grey, respectively. The hydrophobic stacking surface in YTHDC1 can only be aligned by rotating the molecule 180° around the x-axis, suggesting that reader proteins approach RNA from the opposite direction. The m^6^A pocket of METTL3-METTL14 harbors features that enable it to act as an atypical m^6^A sensor/reader during its switch from writer to reader. Dashed lines, h-bonds.

The m^6^A-METTL3-METTL14 structure allowed us to gain valuable insights into how m^6^A writer (METTL3-METTL14), eraser (FTO), and reader (YTHDC1) proteins accommodate m^6^A. We examined their m^6^A pocket in detail (Fig. 4a–c). Despite the lack of obvious resemblance at the protein sequence, domain, and structure levels, we observed high similarity in the interaction networks of m^6^A in METTL3-METTL14 to the binding mode of 6mA in FTO (PDB: 5ZMD) and m^6^A in YTHDC1 (PDB: 4R3I) (Fig. 4). Of note, the purine ring of 6mA in FTO stacks between a hydrophobic amino acid, L109 (the equivalent of M402 in m^6^A), from the top and the backbone atoms of V228, S229, and H231 (the equivalent of T472, G473, and H474 in m^6^A) from the bottom. Interestingly, the *arginine clasp* we found in m^6^A-METTL3-METTL14 is also present in 6mA-FTO. Notably, the side chain of R96 in FTO forms a direct h-bond to *N*^*1*^ of 6mA, while the guanidino group of its R322 residue forms a van der Waals interaction with *N*^*6*^ methyl group, akin to identical interactions by R298 and R471 to stabilize m^6^A in m^6^A-METTL3-METTL14. Stacking interactions that lock the sugar moieties in place also display similarities. For example, the sugar of 6mA in FTO stacks between I85 and H231, whereas the sugar of m^6^A stacks between I400 and H478 of METTL3 (Fig. 4a, b).

We found that m^6^A in METTL3–14 and YTHDC1 (PDB: 4R3I) had many similarities and striking differences, mainly in the orientation of the base (Fig. 4c). The *N*^*1*^ of m^6^A forms an h-bond with N367, whereas an h-bond with carbonyl of S378 akin to carbonyl of R471 of METTL3 stabilizes the *N*^*6*^. Additional hydrophobic interactions from W377 and W428 also support the *N*^*6*^ methyl group in YTHDC1. The nature of stacking interactions for the purine ring is also similar, i.e., hydrophobic residues M434, L380, and L439 on one side and backbone atoms of K361, S362, and N363 on the other. However, the orientation of the m^6^A base in YTHDC1 is reversed by 180° compared to 6mA in FTO and m^6^A in METTL3–14. As such, when the direction of sugars and phosphates of modified bases is aligned in three structures (facing downward in Fig. 4a, b, and upper panel of c), the hydrophobic residues (M434/L380/L439) in YTHDC1 stack from the bottom side and the backbone atoms of K361, S362, and N363 stack from the top side, in contrast to the base orientation in FTO and METTL3. A ~180° rotation of YTHDC1 will place the interacting residues in all three proteins in the same plane. However, the orientation of ribose and phosphate of m^6^A in YTHDC1 will also be reversed (facing upward, Fig. 4c lower panel). Thus, a m^6^A reader protein approaches the m^6^A entirely differently than a writer or eraser. This unique geometric difference may allow the reader to avoid clashes with a writer or eraser enzyme acting simultaneously on the same transcript.

There could be two scenarios for stabilizing m6A in the cryptic pocket: **a.** The pocket may have the capacity to bind to all nucleotides, but m6A can outcompete other nucleotides due to its higher binding affinity to the MTase core. **b**. The pocket may exclusively interact with m6A while nucleotides flanking A/m6A weakly interact with other RNA binding domains of METTL3–14. Consequently, mutations near the pocket could disrupt the binding to m6A or alter the preference from m6A to other nucleotides. We show that METTL3 possesses features that enable it to act as an atypical m^6^A sensor/reader – a function ideally suited for its emerging non-catalytic functions, including crosstalk with eIF3H to promote mRNA circularization, thereby enhancing RNA translation as observed in lung cancer^23,25^ and bone marrow mesenchymal stem cells^21^. Consistently, METTL3 exhibits a strong affinity to a methylated (m^6^A) RNA form, especially those containing secondary structures, a feature that could be important for its non-catalytic roles such as an ‘m^6^A reader’ for an alternative mode of translation initiation during oncogenic translation^23,25^ and cellular stress (e.g., heat shock)^34^.

## Methods

### METTL3-METTL14 MTase core

The gene encoding the MTase domains of human METTL3 (aa 357–580aa) and METTL14 (aa 116–402) were cloned into a pETduet-1 vector and expressed in E. coli NiCo21(DE3) cells. The transformed cells were grown in Terrific Broth medium supplemented with 1 mM ampicillin at 37°C until OD_600nm_ reached 0.6. Protein expression was then induced by adding 0.4 mM isopropyl β-D-thiogalactopyranoside, and the culture was grown at 18°C for 16 hrs. The cell pellets were harvested by centrifugation at 6000 r.p.m. at 4°C and resuspended in cold lysis buffer containing 25 mM Tris pH 8.0, 0.5 M NaCl, 10% glycerol, 5 mM imidazole, 0.1 mM TCEP, one tablet of protease inhibitor (Roche), lysozyme (0.1 mg/mL), and DNase I (5U/mL) and stirred gently at 4°C until achieving full homogeneity. Resuspended cells were lysed by two passages through a microfluidizer (Analytik, UK) and subjected to centrifugation at 41,000 r.p.m. for 50 min at 4°C. The clarified supernatant was filtered through a 0.22 μm filter and loaded onto a Nuvia IMAC column (Bio-Rad) pre-equilibrated with wash buffer (25 mM Tris pH 8.0, 0.5mM NaCl, 10% glycerol, 5 mM imidazole, and 0.1 mM TCEP). The His-tagged METTL3 was co-eluted with untagged METTL14 by increasing the imidazole concentration. The eluates were dialyzed in a buffer lacking imidazole overnight at 4°C in the presence of the ULP1 enzyme to remove the His-SUMO tag from METTL3 proteolytically. The dialyzed proteins were then re-loaded onto an IMAC column to remove un-cleaved proteins and the His-SUMO tag. Two successive passages through MonoQ and Hiload Superdex75 columns (Cytiva) further purified the tag-free complex. The fractions of a homogenous peak eluted in 20 mM Tris pH 8.0, 0.2 M NaCl, and 0.1mM TCEP were pooled, concentrated to 15 mg/ml, and either used immediately or flash-frozen in liquid nitrogen and then stored at −80°C.

### Full-length METTL3-METTL14 and mutants

The full-length human METTL3 and METTL14 (wild-type and mutants) were expressed in insect cells (ExpiSF Expression System, Thermo Fisher) and purified using a protocol published earlier^12^. In brief, the METTL3 and METTL14 plasmids were transformed individually into Max Efficiency DH10Bac competent cells (Thermo Fisher) to generate the DNA bacmids. The successful insertion of genes was confirmed by PCR amplification using a pUC/M13 primer (Forward: 5’- CCCAGTCACGTTGTAAAACG −3’, Reverse: 5’ – AGCGGATAACAATTTCACACAGG −3’). The amounts of purified bacmids and ExpiFectamine SF transfection reagent (Thermo Fisher) were optimized as per the manufacturer’s recommendations (Thermo Fisher). The ExpiSf9 insect cells were cultured in ExpiCD medium (Thermo Fisher) at 125 r.p.m. and 27°C in a non-humidified, air-regulated environment. The cells were harvested 72 hrs post-infection by spinning at 300 × g for 5 min. The PBS-washed cells were resuspended in cold lysis buffer containing 0.5% Igepal, two tablets of protease inhibitor (Roche), and DNase I. Cells were lysed by passing through a microfluidizer (Analytick, UK) and clarified by centrifugation at 41,000 r.p.m. for 40 min.

The proteins were purified using a strategy similar to that of the MTase core, except for removing the His-tag from METTL3. This step was achieved by incubating proteins after the affinity column step with TEV protease for 3 hrs at room temperature. A second passage through a nickel IMAC column removed contaminants and any uncleaved fractions. The complex was then successfully purified by successive passages through HiTrap Heparin and Hiload Superdex 200 columns (Cytiva). Eluates from a homogenous peak of a Superdex column run in a buffer of 0.02 M Tris pH 8.0, 0.15 M NaCl, and 5% glycerol were pooled, concentrated to 1–3 mg/ml, and flash-frozen in liquid nitrogen and stored at −80°C. All full-length METTL3-METTL14 mutants (METTL3: T433A, K459A, R471H, Δ472–473; Δ472–474, H478A, E481A; METTL14: R298P) were generated by site-directed mutagenesis and purified by the same method as the wild-type protein.

### Crystallization, data collection, and structure determination

The crystallization of the human METTL3-METTL14 MTase core (at 10 mg/mL concentration) was carried out by an OryxNano robotic system (Douglas Instruments) using the sitting-drop vapor diffusion method at 20°C. Initial crystals were grown in a solution containing 0.1 M MES pH 6.0, 1.0 M potassium sodium tartrate. After several rounds of optimization by varying pH and salt concentrations, large reproducible crystals were grown in seven days. The *N*^*6*^-methyladenosine monophosphate (m^6^AMP; Sigma, M2780) was soaked into native crystals (2.0 mM concentration) for 1 hr at 20°C. A complete diffraction dataset was measured to ~2.5Å at GMCAT 23ID-D beamline at Advanced Photon Source, Chicago, IL. The apo structure of METTL3-METTL14 MTase core (PDB: 5IL0) was used as a search model for molecular replacement in Phenix^45^. The structure was iteratively built and refined using Coot (Version 0.9.8.6)^46^, Phenix (Version 1.15.2–3472) and Buster (Version 2.10.4)^47^, respectively. The ligand geometry restraints were generated by Grade. All structure figures were generated using Pymol (Schrodinger Suite).

### *In vitro* methyltransferase assays

5 μM [methyl-^3^H] SAM (PerkinElmer), 10 μM substrate RNA (NEAT2*: 5’ – GCCUAGUAGCAGAGAGGACUGCUCCUUGGU - 3’), and 2 μM purified WT or mutated METTL3-METTL14 were mixed and incubated at 37°C for 1 hr in a total volume of 5 μL in a reaction buffer (50 mM HEPES pH 7.5, 5 mM NaCl, and 1 mM DTT). The reactions were quenched by blotting 3 μL of each on the Hybond-N+ membrane (Amersham). The methylated substrates were then crosslinked by exposing them to ultraviolet light (254 nm) for 2 min. The membranes were washed three times with 1X PBS, followed by two 95% ethanol washes. Then the membranes were air-dried inside the hood for 15 minutes, and the RNA probe’s count per minute (c.p.m.) on each membrane were measured by a scintillation counter (Beckman LS6500). All results are reported as the means from three independent experiments (n=3) for each group, with one standard deviation (s.d.).

### Fluorescence polarization

The reactions were carried out in a buffer containing 10 mM HEPES pH 7.5 and 50 mM KCl. The two 30-mer RNA probes (native RNA or A-RNA and its m^6^A-modified version or m^6^A-RNA) were synthesized with a fluoresceine moiety covalently attached to their 5’-end, de-protected, and purified using HPLC (HorizonDiscovery). The sequence of A-RNA was identical except the target A base within the characteristic motif (underlined and bold) in native A-RNA (Fl-NEAT2*: 5’ – [Fl]GCCUAGUAGCAGAGAGG**A**CUGCUCCUUGGU - 3’), was replaced by *N*^*6*^methyladenosine (m^6^A) in the modified RNA (Fl-NEAT2*-m^6^A: 5’ – [Fl]GCCUAGUAGCAGAGAGG**[m**^**6**^**A]**CUGCUCCUUGGU - 3’). A constant 5 nM of RNA probes were incubated with increasing concentrations of the purified WT or mutant METTL3-METTL14 enzymes in a 384-well plate. The fluorescence polarization values (excitation wavelength = 485 nm, emission wavelength = 530 nm) of each reaction were measured by PHERAstar FS (BMG Labtech). The affinity of RNA-protein binding was calculated by a simple one-site specific binding model (Y = Bmax*X/(Kd+X), X = protein concentration, Y = specific binding, Bmax = maximum specific binding, Kd = equilibrium dissociation constant). The results were analyzed and fitted by GraphPad Prism (GraphPad Software, San Diego, CA). Each experiment was repeated three times independently (n=3), and final *K*_*d*_ is reported as the mean of the three replicates with standard deviation (s.d.) for each RNA shown as error bars.

### Steady-state fluorescence assays

For this experiment, we used a 14-mer single-stranded RNA probe in which the the target adenine base within the m^6^A motif was replaced with 2-aminopurine (2-Ap) (r6T*: 5’ – CUUCGG[2-Ap]CUCUGCU – 3’). In a 384-well plate format, 0.5 μM of RNA probe mixed with increasing concentrations (1 – 5 μM) of full-length human WT or mutant METTL3-METTL14 enzymes in a 20 μL reaction in the buffer containing 50 mM Tris pH 8.0, and 10 mM MgCl_2_ and incubated at room temperature. The reaction was excited at 320 nm with a 325 nm cut-off wavelength in a SpectraMax M5 microplate reader (MolecularDevices). The fluorescence emission was measured at 371 nm and 37°C every 5 minutes from 0 – 60 minutes and then every 30 minutes until the end time point (120 minutes). The data were analyzed and fitted by GraphPad Prism (GraphPad Software, San Diego, CA) using the Michaelis-Menten model (Y=Vmax*X/(Km+X), X = protein concentration, Y = enzyme velocity, Vmax = maximum enzyme velocity, Km = Michaelis-Menten constant). All results reported are mean values from three independent experiments with standard deviations (s.d.) shown as error bars.

### Surface plasmon resonance (SPR)

The surface plasmon resonance experiments were performed using a Biacore 1S+ equipped with a CM5 sensor chip. Recombinant streptavidin (Millipore Sigma, 11721674001) was first immobilized at all six flow cells using amine-coupling chemistry. The surfaces of flow cells were activated for 7 min with a 1:1 mixture of 0.1 M NHS (N-hydroxysuccinimide) and 0.4 M EDC (3-(N, N-dimethylamino) propyl-N-ethylcarbodiimide) at a flow rate of 10 μl/min. The streptavidin at a concentration of 50 μg/ml in 10 mM sodium acetate, pH 5.5, was immobilized at a density of around 6,000 RU on all six flow cells. All surfaces were blocked with a 7-minute injection of 1 M ethanolamine, pH 8.0. The biotinyl-RNA substrates were then diluted to 100nM in running buffer (10mM HEPES, 150mM KCl, 0.05% P20, pH 7.5) and captured in flow cell 3–6 respectively (12s, 5 μl/min) to reach a level around 50RU (see details below). To measure kinetic binding data, the analytes (METTL3-METTL14_WT_, METTL3-METTL14_R298P_, and METTL3-METTL14_R471H_ proteins) were diluted in the same running buffer, with five concentrations ranging from 2.4 to 1500 nM. The analytes were then injected over all flow cells at various concentrations in single-cycle kinetics format at a flow rate of 30 μl/min at 25°C. The analyte was allowed to associate and dissociate for 120 seconds for each injection and dissociate for 1200 seconds, respectively. Data were collected at a rate of 10 Hz. The data were fit to a 1:1 binding model or two-state reaction model, as mentioned, using the data analysis option available within Biacore Insight Evaluation Software (Version 5.0.18.22102).

### Molecular Dynamics Simulations

#### Structure preparation

The crystal structure of the METTL3-METTL14 complex bound to M^6^A was used as the starting point for all classical molecular dynamics simulations. The AlphaFold model of the METTL14 subunit (UniProt identifier: AF-Q3UIK4-F1) was integrated into the original crystal structure, filling in the gaps and creating a refined METTL3-METTL14 enzyme structure that accounted for the missing residues. Mutants T433A, K459A, R471H, H478A, E481A, and R298P were generated using ICM-Pro software (www.molsoft.com). Additionally, the Δ472–473 and Δ472–474 deletions were obtained from the AlphaFold model. The refined structure was aligned to the crystal structure (PDB ID: 7CV6), and the position of S-adenosyl-L-homocysteine was identified as the initial position of m^6^A for the m^6^A-bound METTL3-METTL14 complex. The ProteinPrepare module implemented in the PlayMolecule was employed to assign the protonation states of residues at pH 7.0^48^.

#### System setup and simulation protocol

The simulations were conducted using the Amberff14SB force field for the protein^49^. The m^6^A was subjected to geometry optimization using Gaussian 16 (HF/631G*) (www.gaussian.com). The m^6^A parameters were derived with GAFF as implemented in Ambertools23 using antechamber and parmchk tools^50^. RESP partial charges were calculated with Gaussian 16 following the procedure suggested by antechamber^51^. The preprocessed structure was explicitly solvated in a cubic periodic of water molecules represented by the transferrable intermolecular potential with 3 points (TIP3P), whose boundary is at least 10 Å from any solute atoms so that the protein does not interact with its periodic images. Periodic boundary conditions in all directions were utilized to reduce the finite system size effects. To neutralize the total charge, Na^+^/Cl^−^ counterions were added. Subsequently, the systems were energy minimized by 5000 steps with the conjugate gradient method to remove any local atomic clashes. Initial velocities within each simulation were sampled from Boltzmann distribution at a temperature of 300 K. The solvents were equilibrated for five ns under the NPT ensemble. The production simulations of supervised molecular dynamics were run under the NVT ensemble using a Langevin thermostat with a damping of 0.1 ps^−1^ and hydrogen mass repartitioning scheme to achieve time steps of 2 fs. Berendsen thermostat and Langevin barostat were used to keep the temperature and pressure constant, respectively^52^. Long-range electrostatic interactions were computed using the particle mesh Ewald summation method^53^. The cutoff radius for neighbor searching and nonbonded interactions was taken to be 9 Å with a switching distance of 7.5 Å was used, and all bonds were constrained using the LINCS algorithm^54^. In total, > 20 SuMD simulations were run as a swarm. Of these, the first three replicas that met the supervision criteria were selected for analysis. All simulations were run using the ACEMD engine^55^.

#### Supervision procedure

Supervised molecular dynamics is a method that can accelerate the binding process between ligands and protein recognition without introducing bias^56^. This method employs an algorithm that monitors the distance between the ligand and the protein. Short simulations of specific lengths are run, and the distance between the ligand and the protein is calculated. The fitting of linear least squares is applied to fit the data, showing the distance against time. If the slope of the resulting straight line is negative, indicating that the ligand is approaching the binding site, then the state of the last frame, including the velocity and the coordinates of this short trajectory, will be used as the initial state for the next short trajectory. On the contrary, if the slope is positive, thereby indicating that the ligand is not approaching the binding site, the short trajectory will be discarded, and the simulation will restart from the last initial state. However, to avoid the ligand being stuck, assessed by 10 consecutive failed steps, a relatively longer simulation is run, followed by the final state of the simulation being used as the starting point for the successive step. The supervision algorithm is switched off when the distance reaches a defined threshold value. To explore the m^6^A swivel from substrate A binding pocket to product m^6^A binding pocket, a supervision protocol was designed in two steps. In the first step, during the production run of the MD trajectory, the distance between the center of mass of the m^6^A and the center of mass of the residues constituting the product m^6^A binding pocket (METTL3: I400, M402, T433, G434, R435, R471, T472, G473, H474, H478, E481, and METTL14: R298) is monitored over a fixed time window (0.02ns). In the second step, the distances between two key atom pairs were calculated, namely R298:NH1-m^6^A:N1 and H478:ND1-m^6^A:P1. The trajectory analysis was carried out, and the figures were made using PyMol (www.pymol.org) and VMD^57^. The graphs were made using Python scripts.

## Figures and Tables

**Table 1.** Capture level of different RNA substrates

Flow cell	RNA substrate	Captured (RU)

3	rNEAT2	66.4
4	rNEAT2-m6A	62.7
5	r6T	48.9
6	r6T-m6A	51.2

**Table 2.** Kinetic parameters for all evaluated bindings with 1:1 binding model

Ligand RNA	Analyte Protein	K_on_ (10^5^ M^−1^ S^−1^)	K_off_ (10^−2^S^−1^)	K_D_ (nM)	Rmax (RU_max_)

rNEAT2	METTL3-METTL14	1.64	4.22	256	5.19×10^2^
rNEAT2-m6A	METTL3- METTL14	0.755	2.55	337	4.50×10^2^
r6T	METTL3- METTL14	0.855	11.6	1360	4.21×10^2^
r6T-m6A	METTL3- METTL14	0.0774	24100	3120	5.55×10^2^

**Table 3.** Kinetic parameters for all evaluated bindings with two state reaction model

Ligand RNA	Analyte Protein	K_on1_ (M^−1^ S^−1^)	K_off1_ (S^−1^)	K_on2_ (M^−1^ S^−1^)	K_off2_ (S^−1^)	K_D_(nM)	Rmax (RU_max_)

rNEAT2	R471H	2.59×10^4^	2.17×10^−2^	6.02×10^−3^	4.54×10^−4^	58.8	6.97×10^2^
	R298P	2.93×10^4^	2.28×10^−2^	3.68×10^−3^	3.11×10^−3^	356	3.73×10^2^
rNEAT2-m6A	R471H	2.73×10^4^	2.22×10^−2^	5.82×10^−3^	4.85×10^−4^	62.8	5.03×10^2^
	R298P	2.38×10^4^	2.77×10^−2^	6.09×10^−3^	5.23×10^−3^	538	3.23×10^2^
r6T	R471H	4.43×10^4^	3.85×10^−2^	5.06×10^−3^	7.21×10^−4^	108	4.19×10^2^
	R298P	5.38×10^3^	4.00×10^−2^	2.14×10^−3^	8.79×10^−4^	2160	5.52×10^2^
r6T-m6A	R471H	6.87×10^6^	8.99	6.74×10^−3^	1.01×10^−3^	171	2.70×10^2^
	R298P	6.62×10^2^	3.66×10^−2^	2.09×10^−3^	1.48×10^−3^	22900	2.47×10^3^

## Data Availability

The coding sequences of METTL3 (NCBI reference sequence GI: 33301371) and METTL14 (NCBI reference sequence GI: 172045930) used in this study are available at NCBI. We have provided source data as a separate Source Data file. The atomic coordinates and structure factors were deposited in the Protein Data Bank (PDB) under accession code 9DGJ (m6AMP) and DOI: 10.2210/pdb9dgj/pdb. The start files and the trajectories from molecular dynamics simulations can be downloaded from https://doi.org/10.5281/zenodo.12681486. Requests for additional material and information should be directly addressed to Y.K.G. (guptay@uthscsa.edu).
