# Supplementary material for: A cryptic pocket in METTL3-METTL14 regulates m6A conversion and sensing
Source: Res Sq. 2023 Aug 8:rs.3.rs-3150186. Preprint. [Version 1] doi: 10.21203/rs.3.rs-3150186/v1 (PMC10441475; doi:10.21203/rs.3.rs-3150186/v1)
Supplement: Supplementary file 1 [file NIHPPrs3150186v1-supplement-1.pdf]

Supplementary Files

This is a list of supplementary files associated with this preprint. Click to download.

- ExtendeddataNature070723.pdf
- m6amoviecaptionthinnerarrow.mov
- D1000275686valreportfullP1.pdf
